# DLKcat cannot predict meaningful *k*_cat_ values for mutants and unfamiliar enzymes

**DOI:** 10.1093/biomethods/bpae061

**Published:** 2024-08-24

**Authors:** Alexander Kroll, Martin J Lercher

**Affiliations:** Institute for Computer Science and Department of Biology, Heinrich Heine University, D-40225, Düsseldorf, Germany; Institute for Computer Science and Department of Biology, Heinrich Heine University, D-40225, Düsseldorf, Germany

**Keywords:** DLKcat model, DLKcat limitations, kcat, kcat prediction, enzyme turnover number, deep learning, sequence similarity, machine learning limitations, protein sequence identity, data splitting

## Abstract

The recently published DLKcat model, a deep learning approach for predicting enzyme turnover numbers (*k*_cat_), claims to enable high-throughput *k*_cat_ predictions for metabolic enzymes from any organism and to capture *k*_cat_ changes for mutated enzymes. Here, we critically evaluate these claims. We show that for enzymes with <60% sequence identity to the training data DLKcat predictions become worse than simply assuming a constant average *k*_cat_ value for all reactions. Furthermore, DLKcat’s ability to predict mutation effects is much weaker than implied, capturing none of the experimentally observed variation across mutants not included in the training data. These findings highlight significant limitations in DLKcat’s generalizability and its practical utility for predicting *k*_cat_ values for novel enzyme families or mutants, which are crucial applications in fields such as metabolic modeling.

## Results 

### DLKcat cannot provide meaningful predictions for wildtype enzymes dissimilar to training enzymes and for mutants not included in the training data

The turnover number *k*_cat_ quantifies the catalytic efficiency of enzymes. As experimental *k*_cat_ estimates are expensive and time-consuming, it is desirable to develop computational pipelines that can predict turnover numbers of arbitrary enzymes from easily accessible features. Advances in deep learning have now put such predictions into reach [1, 2]. In a recent publication, Li *et al*. [3] described DLKcat, a general deep learning model for *k*_cat_ predictions. The authors state that their approach facilitates “high-throughput *k*_cat_ prediction for metabolic enzymes from any organism merely from substrate structures and protein sequences.” Furthermore, they claim that “DLKcat can capture *k*_cat_ changes for mutated enzymes”. Here, we show that DLKcat predictions are accurate only for enzymes that are highly similar to proteins used for training and become worse than simply assuming a constant average *k*_cat_ value for all reactions for enzymes without close homologs in the training data. We further show that DLKcat’s mutant predictions—all of which were made for enzymes highly similar to training data—are much less accurate than implied by the DLKcat publication, capturing none of the experimentally observed variation across mutants not included in the training data.

The performance of machine learning predictions for enzyme features depends strongly on the sequence similarity between a target enzyme and enzymes in the training set [4], consistent with the widely held notion that enzymes with more similar amino acid sequences are more likely to be functionally similar [5]. Accordingly, it is likely much easier to predict unknown *k*_cat_ values for enzymes used in model training than to make predictions for enzymes that have no close homologs with known kinetic constants. More than two-thirds (67.9%) of the enzymes in the DLKcat test set are also included in the training data, and an additional 23.3% have amino acid sequences that are at least 99% identical to sequences in the training data. This extremely high similarity between training and test data constitutes a central problem in the construction of DLKcat [6], given that its authors aimed to generate a prediction model that generalizes well to “enzymes from any organism”—that is, to proteins with amino acid sequences that may often differ by more than a few percent from those in the training data.

**Figure 1.**
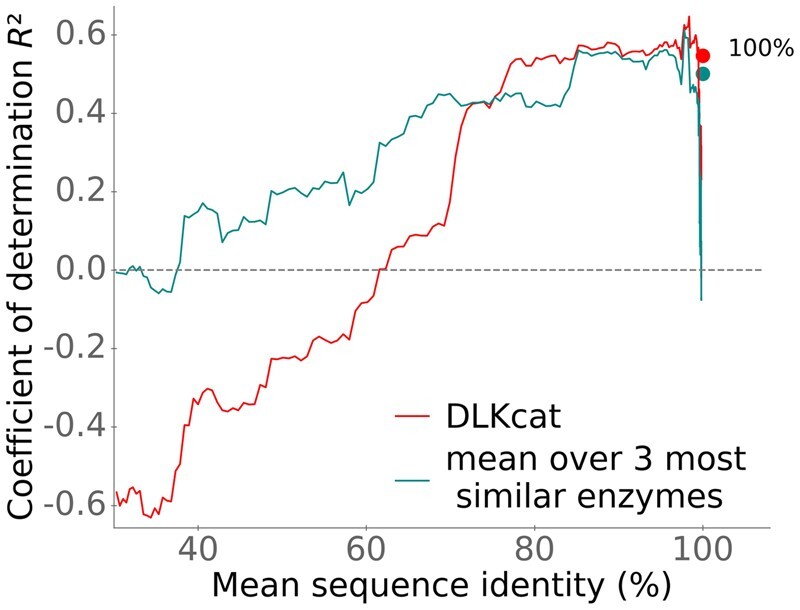
DLKcat predictions become reasonable only when closely related enzymes were used for training (max. sequence identity > 70%) and are barely better than simple *k*_cat_ averages even when the same enzyme was used for training. The curves are coefficients of determination *R*^2^, calculated in sliding windows of size *n* = 100 across sequences in the test set ordered by the maximal sequence identity between individual test enzymes and all sequences in the training data. Position on the *x*-axis indicates the mean across the window. Eighty-two test data points fall in the range of 0%–40% sequence identity, 42 data points fall in the range of 40%–80%, 27 data points fall in the range of 80%–99%, and 1536 data points fall in the range of 99%–100%. DLKcat predictions are shown in red. For comparison, the cyan line shows the geometric mean of *k*_cat_ values, calculated over the three most similar enzymes in the training set (without considering the catalyzed reactions or substrates; *R*^2^ = 0.420 of the mean approach vs. *R*^2^ = 0.445 for DLKcat, calculated across the complete test dataset, *N* = 1687). The filled circles at the top right are for test data points with enzymes already used for training (100% max. sequence identity, *N* = 1143); these were not included in the sliding windows.

The red line in Fig. 1 shows how DLKcat’s prediction quality depends on the sequence identity between an enzyme in the test dataset and the most similar enzymes used for training. The figure shows sliding window estimates of the coefficient of determination, *R*^2^, which is a widely used measure for prediction quality (see Equation (2) in Li *et al*. [3]). *R*^2^ = 1 indicates perfect predictions. As seen from Fig. 1, DLKcat’s coefficients of determination are negative for maximal sequence identities between test and training data below 60%. This indicates that when no close homologs have been used for training, DLKcat predictions are typically worse than simply assuming the same mean *k*_cat_ value for all reactions, which corresponds to *R*^2^ = 0.

**Figure 2.**
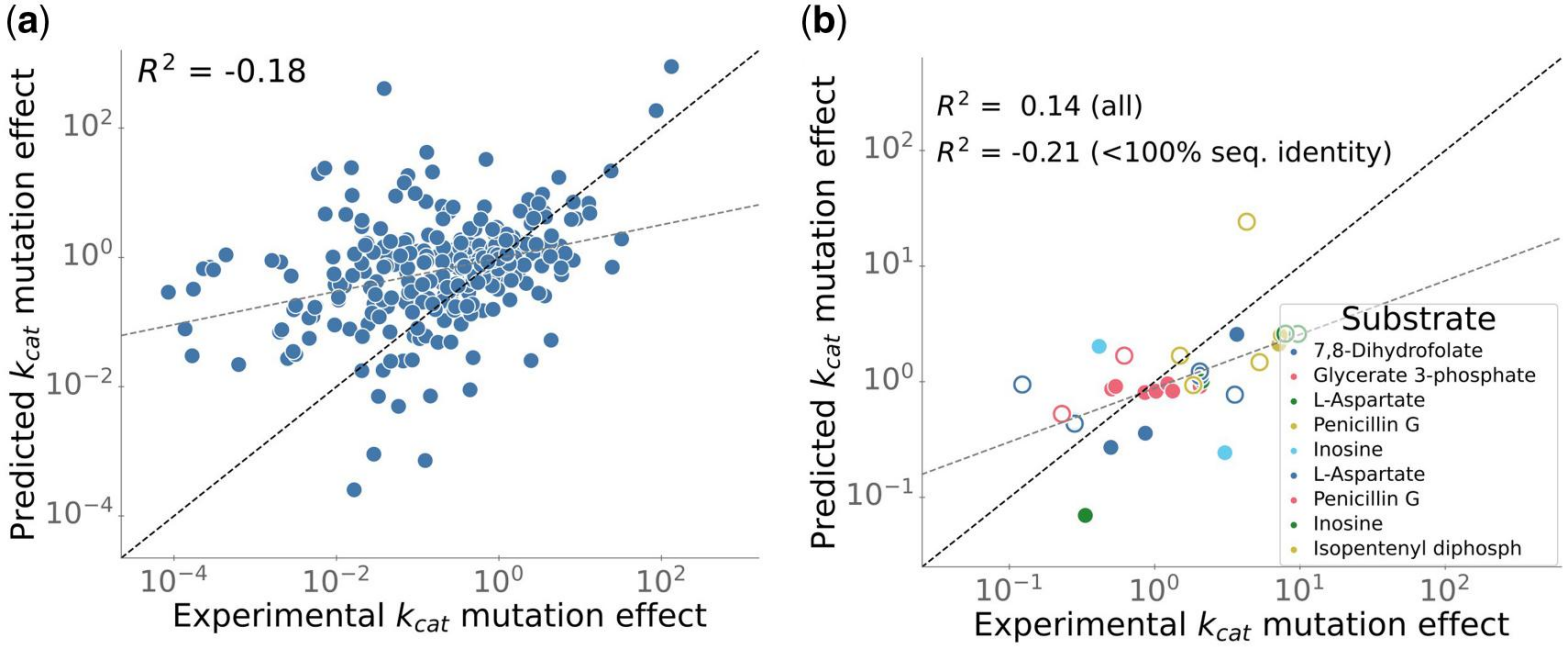
DLKcat predicts only a small fraction of *k*_cat_ variation due to mutations even for mutants that are highly similar to proteins used for training. (**a**) For all mutants in the test set with maximum sequence identities above 99% and below 100% compared to sequences in the training set, we compared predicted to experimentally observed values. To allow a global analysis of mutation effects on *k*_cat_, we scaled the log10-transformed *k*_cat_ values to mutation effects as follows. For each enzyme-substrate pair, we calculated the mean across all measured *k*_cat_ for all other enzymes with the same substrate and with sequence identities above 98%; from each measured and each predicted *k*_cat_ for this subset, we then subtracted this mean. **(b)** For all mutants in Fig. 3c of Ref. [3], we compared predicted and experimentally observed changes in *k*_cat_ due to the mutations. Only data points from the test dataset are shown. Colors indicate different enzyme–substrate pairs. Open circles are mutants identical to enzymes in the training data, while solid dots are mutants with between 99.4% and 99.8% sequence identity to enzymes in the training data. We scaled the log10-transformed *k*_cat_ values to *k*_cat_ mutation effects in a similar manner as described for panel (a). For each enzyme–substrate pair, we calculated mean across all measured *k*_cat_ for the wild-type and the different mutants in the complete dataset; from each measured and each predicted *k*_cat_ for this enzyme-substrate pair, we then subtracted the mean. The grey dashed lines show the line of best fit.

When attempting to predict the effects of mutations, DLKcat is even less able to generalize beyond the proteins used for training. To support DLKcat’s ability to predict mutation effects, Li *et al*. calculated a combined Pearson correlation across the measured and predicted *k*_cat_ values for mutants of different enzyme-substrate pairs. However, since different enzymes can have widely different *k*_cat_ values, simultaneously comparing *k*_cat_ predictions for mutants of different enzymes would lead to strong correlations even for a prediction model that simply outputs the *k*_cat_ value of the corresponding wildtype for all mutants, as long as the mutant *k*_cat_ values are distributed around the corresponding wildtype values. To assess mutation effects, we need to focus instead on the differences between the *k*_cat_ of closely related variants of the same enzyme. Thus, to facilitate a global analysis of mutant prediction quality, we grouped all *k*_cat_ values of enzymes with over 99% sequence identity and with the same substrate—including mutants and wildtypes—and computed for each mutant the difference of its log10-transformed *k*_cat_ value from the geometric mean of the corresponding group (see Fig. 2 caption for details). We refer to this value as the *k*_cat_ mutation effect. 385 out of 744 mutants in the test dataset were also part of the training set, so they are not informative with respect to DLKcat’s ability to extrapolate beyond the proteins used for training. Except for five enzymes, all others have at least 99% amino acid sequence identity (*N* = 354) to sequences in the training set. Although these mutants are almost identical to proteins used for training, DLKcat cannot successfully predict the mutation effects on *k*_cat_, indicated by a negative coefficient of determination (*R*^2^ = −0.18; Fig. 2a).

Arguably, the most striking result presented in Ref. [3] is its Fig. 3c, which presents a “comparison between predicted and measured *k*_cat_ values for several well-studied enzyme-substrate pairs with rich experimental mutagenesis data”. The panel is augmented with a Pearson correlation coefficient *r* = 0.94 and corresponding *P*-value <10 ^− 91^, suggesting that DLKcat can accurately predict the effects of mutations on *k*_cat_. However, only 15.6% of the data in this panel had been set aside for testing, while 71.9% of the data points shown were part of the training data. The remaining 12.5% of the data points were part of the validation data, which was used to select the hyperparameters of the prediction model. Any meaningful analysis of DLKcat’s abilities must be restricted to data from the test set, as done in Fig. 2b, which again uses *k*_cat_ mutation effects to provide a more appropriate presentation of the Fig. 3c of Li *et al*. [3] data. Fourteen of the 30 mutant enzymes in the test set had also been used for training, and each of the remaining 16 mutant amino acid sequences is at least 99.4% identical to a sequence in the training set. Despite this close relationship between test and training enzymes, DLKcat predicts only a small fraction of the variation in *k*_cat_ due to mutations: when calculating a coefficient of determination across the *k*_cat_ mutation effects, one obtains a low coefficient of determination, *R*^2^ = 0.14. When restricting this analysis to the 16 mutants that were not in the training data, the coefficient of determination is further reduced to *R*^2^ = −0.21. Thus, for previously unseen mutants of the enzyme-substrate pairs in Li *et al*.’s [3] Fig. 3c, DLKcat predicts none of the variance relative to the wildtype *k*_cat_, even though these mutants are highly similar to wild-type and/or mutant enzymes in the training data.

In sum, while Li *et al*. claim that DLKcat can predict *k*_cat_ for metabolic enzymes from any organism and can capture *k*_cat_ changes for mutated enzymes, the above analyses demonstrate that these claims are inconsistent with a careful analysis of the data in Ref. [3]. For enzymes without close homologs in the training data (<60% sequence identity), DLKcat predictions are worse than simply assuming a constant average *k*_cat_ value for all reactions (*R*^2^ < 0, Fig. 1), and DLKcat makes no meaningful predictions for mutants not included in the training set. Thus, DLKcat provides little benefit when predicting turnover numbers for enzyme families and mutants not already characterized kinetically, which arguably constitute the most important use cases in most applications, including the parameterization of enzyme-constrained genome-scale metabolic models (ecGEMs).

### Overcoming the limitations of DLKcat

The disappointing performance of DLKcat can be attributed, at least in part, to the construction of its datasets and the training, validation, and test splits, which did not challenge the model to predict *k*_cat_ values for enzymes other than those in the training set. In many cases, the DLKcat dataset contains multiple measurements for a single enzyme–substrate pair, mostly by combining a wild-type enzyme with its mutants. Randomly partitioning such a dataset into training, validation, and test sets will typically result in at least one measurement for an enzyme–substrate pair being in the training set and at least one other measurement for the same enzyme-substrate pair being in the validation and/or test set. This problem was exacerbated by the fact that DLKcat considered enzyme–substrate pairs instead of enzyme–reaction pairs. For multi-substrate reactions, this resulted in multiple data points for the exact same *k*_cat_ measurement, that is, the same *k*_cat_ measurement can be present in the training set and the validation or test set.

As a result, more than two-thirds of the enzymes in the DLKcat test set were also present in the training data, and more than 90% of the test sequences had an amino acid identity of at least 99% to at least one sequence in the training data. The same is true for the validation set, since it was generated in the same way as the test set. Such a data structure can be very problematic because the model architecture and model hyperparameters are chosen to achieve high performance on the validation set. However, if the validation dataset consists mostly of enzymes that are identical or nearly identical to the training enzymes, a model will be selected that simply remembers the training data. Such a model is not forced to learn the underlying rules needed to predict *k*_cat_ values for unseen enzymes. As a result, DLKcat cannot predict *k*_cat_ values for enzymes that have less than 60% maximum sequence identity to the training enzymes.

It has been shown that it is possible to achieve successful *k*_cat_ prediction models from similar experimental data using only enzyme sequence information and information about the molecular structure of the reactants [1, 2]. To overcome the limitations of DLKcat, it is important to ensure that the validation split does not consist primarily of enzymes that are identical or nearly identical to those in the training set. This can be achieved by grouping enzyme–substrate pairs that belong to the same enzyme and, for most groups, ensuring that all pairs from a group are assigned to a single split. An alternative splitting approach is to use the cd-hit algorithm to compute clusters of enzyme sequences that are then assigned to the same fold, where sequences from different clusters do not exceed a certain similarity threshold [7].

The issue of multiple data points belonging to the same measurement could be addressed by constructing enzyme–reaction pairs instead of enzyme–substrate pairs, although this will require additional data annotation and curation. At a minimum, data points belonging to the same *k*_cat_ measurement should be placed in the same split to avoid data leakage between sets.

## Materials and methods

To reproduce the DLKcat model and to make predictions for the corresponding test set, we downloaded the code provided on GitHub by Li *et al*. [3]. To calculate the maximal amino acid sequence identity between each enzyme in the test set and all enzymes in the training set, we used the Needleman–Wunsch algorithm implemented in the EMBOSS software package [8].

## Data Availability

All software was coded in Python 3. The code used to generate the results of this article, in the form of Jupyter notebooks, as well as all datasets, are available from https://github.com/AlexanderKroll/DLKcat_Analysishttps://github.com/AlexanderKroll/DLkcat_Matters_Arising.
